# Editorial: Glioma: from genetic to cellular heterogeneity

**DOI:** 10.3389/fcell.2024.1403595

**Published:** 2024-04-16

**Authors:** K. A. Walentynowicz, L. F. Stead, A. Ellert-Miklaszewska, K. Karakoula

**Affiliations:** ^1^ Cancer Biology and Genetics, Memorial Sloan Kettering Cancer Center, New York, NY, United States; ^2^ Leeds Institute of Medical Research, St James’s University Hospital, University of Leeds, Leeds, United Kingdom; ^3^ Laboratory of Molecular Neurobiology, Neurobiology Center, Nencki Institute, Polish Academy of Sciences, Warsaw, Poland; ^4^ School of Pharmacy, Faculty of Science and Engineering, University of Wolverhampton, Wolverhampton, United Kingdom

**Keywords:** glioma, heterogeneity, transcriptome, editorial, GBM

Gliomas are primary tumors of the central nervous system that affect individuals of all ages. Staggering 5-year survival rates of 5%–10% for the most aggressive high-grade glioma, glioblastoma, led to intensive research on tumor biology and the microenvironment (Miller et al., 2021; Girardi et al., 2023; Schaff and Mellinghoff, 2023).

Recent advances in research techniques revealed the highly heterogenous nature of these tumors and complex interactions of all cells within the tumor microenvironment (Chen and Hambardzumyan, 2018; Neftel et al., 2019; Varn et al., 2022; Walentynowicz et al., 2023).

A wealth of transcriptomic, genetic, proteomic, and clinical information has been made available via public databases such as The Cancer Genome Atlas (TCGA) or The National Cancer Institute’s Clinical Proteomic Tumor Analysis Consortium (CPTAC), providing valuable resources for data analysis and identification of novel signatures relevant to human cancers, including glioma. This Research Topic includes three original research articles and two reviews covering broad Research Topic of glioma biology. Emerging Research Topic in 7-methylguanosine (m7G) regulators and their association with cancer, immunogenic cell death mechanisms, and senescence in glioma were studied to identify transcriptomic signatures.

Wang et al. reports a novel signature of m7G regulators that correlates with the immune microenvironment and carries prognostic value in high-grade and low-grade glioma patients. Using selected differentially expressed m7G regulators in high-grade glioma (*NUDT5*, *EIF4E1B*, and *NUDT11*) and low-grade glioma (*CYFIP1*, *CYFIP2*, *EIF3D*, *GEMIN5*, *NUDT5*, *NUDT1*, and *EIF4E3*), a prognostic signature was computed. *EIF4E1B* expression was found to correlate with alternative splicing events in both high- and low-grade tumors. Higher expression of immune checkpoint genes and HLA genes was associated with the high-risk group in both tumor grades, following application of the m7G signature.

Cai et al. found immunogenic cell death (ICD)-associated biomarkers that correlate with survival, immune score, and treatment response in low-grade glioma. ICD genes were found to be significantly upregulated in low-grade gliomas compared to normal brain samples. Gene signatures associated with ICD were calculated by GeneMANIA and subsequent clustering identified groups that significantly associated with immune pathway enrichment, immune score, and clinicopathological features. Both ICD-low and high groups harbored high incident of somatic mutations in *IDH1*, *TP53* and *ATRX*, with higher incident of *CIC* (26.3% vs. 13.2%) in ICD-low and *TTN* in ICD high (16.7% vs. 9.5%). The ICD-high group has poorer prognosis but showed higher predicted drug sensitivity compared to the ICD-low group.

Li et al. looked at senescence-related genes in low- and high-grade gliomas, identifying six genes (*AURKA*, *CENPA*, *LIMK1*, *PATZ1*, *TGFB1I1*, *TLR3*) associated with significant survival benefit that could be used in a regression model to predict prognostic ‘risk’ in low-grade glioma. The high-risk group showed enrichment in major glioma over-represented pathways like TNFα signaling via NFκB, epithelial mesenchymal transition, IL6/JAK/STAT3 pathway according to Gene Set Enrichment Analysis. Tumors with a high senescence score were enriched, overall, for immune-cell marker genes, suggesting higher immune infiltration, and had higher expression of immune checkpoint genes like *PD1*, *PDL1,* and *TIM3*. The authors then validated their senescence signature in various glioma samples containing public datasets, confirming that lower senescence signature risk scores significantly associated with survival benefit.

Lastly, two review articles focused on epigenetic dysregulation in glioma. Liu et al. described the impact of long non-coding RNAs (lncRNAs) in glioma progression, as multiple lncRNAs are identified as aberrantly expressed in malignant tumors. Groves and Cooney focused on epigenetic modifications identified in pediatric high-grade glioma and epigenetic therapeutic modalities.

In summary, this Research Topic encompasses focused analysis on gene expression signatures in glioma with potential clinical impact, stratifying patients into various subgroups. It provides an overview of the current state of epigenetic modifying therapies for pediatric gliomas, and the impact of lncRNAs on glioma progression.
